# Comparison of Artificial Intelligence based approaches to cell function prediction

**Published:** 2020

**Authors:** Sarala Padi, Petru Manescu, Nicholas Schaub, Nathan Hotaling, Carl Simon, Kapil Bharti, Peter Bajcsy

**Affiliations:** a ITL, National Institute of Standards & Technology, Gaithersburg, MD, USA; b National Eye Institute, NIH, Bethesda, MD, USA; c MML, National Institute of Standards & Technology, Gaithersburg, MD, USA

**Keywords:** Cell segmentation, Cell function prediction, Retinal Pigment Epithelium Cell, Deep learning, Age-related macular degeneration, Trans-Epithelial Resistance, Vascular Endothelial Growth Factor

## Abstract

Predicting Retinal Pigment Epithelium (RPE) cell functions in stem cell implants using non-invasive bright field microscopy imaging is a critical task for clinical deployment of stem cell therapies. Such cell function predictions can be carried out using Artificial Intelligence (AI) based models. In this paper we used Traditional Machine Learning (TML) and Deep Learning (DL) based AI models for cell function prediction tasks. TML models depend on feature engineering and DL models perform feature engineering automatically but have higher modeling complexity. This work aims at exploring the tradeoffs between three approaches using TML and DL based models for RPE cell function prediction from microscopy images and at understanding the accuracy relationship between pixel-, cell feature-, and implant label-level accuracies of models. Among the three compared approaches to cell function prediction, the direct approach to cell function prediction from images is slightly more accurate in comparison to indirect approaches using intermediate segmentation and/or feature engineering steps. We also evaluated accuracy variations with respect to model selections (five TML models and two DL models) and model configurations (with and without transfer learning). Finally, we quantified the relationships between segmentation accuracy and the number of samples used for training a model, segmentation accuracy and cell feature error, and cell feature error and accuracy of implant labels. We concluded that for the RPE cell data set, there is a monotonic relationship between the number of training samples and image segmentation accuracy, and between segmentation accuracy and cell feature error, but there is no such a relationship between segmentation accuracy and accuracy of RPE implant labels.

## 1. Introduction

Age-related macular degeneration (AMD) is a disease that affects the eye macula. There are 10 million people in the United States of America diagnosed with AMD and the occurrence of AMD is more likely for people over 50 years of age. AMD disease is caused by the death of Retinal Pigment Epithelium (RPE) cells in an eye retina [2,8,29]. RPE cells form a single layer with pigment granules, have tight junctions, and appear to have a hexagonal shape in a healthy implant [15,35]. These visual signs of healthy RPE cells have been shown to be the key qualitative attributes during the 155 day long bio-manufacturing process of RPE cell implants [12,15].

Before a cell implant is delivered to a patient, it must be evaluated for healthy cell function during the implant preparation. Several biological studies have related cell shapes to the implant “quality” [12]. Based on these studies, the microscopy imaging community has been developing supervised and unsupervised automatic methods for RPE cell segmentation as the segmentation can be useful for 1) shape analysis, 2) discrimination of cell regions that are healthy or unhealthy, and 3) measurements of cell count and density [9,23].

In addition to cell shape measurements, Trans-Epithelial Resistance (TER) and Vascular Endothelial Growth Factor (VEGF) measurements have been used for assessing the health of RPE cell implants. TER is a quantitative technique to measure the integrity of tight junction dynamics in cell culture models of epithelial monolayers of an eye retina. The ranges of TER and VEGF values can be indicators of healthy (TER *>* 400 Ω*:cm*^2^, VEGF ratio *>* 3) or unhealthy (TER *<* 400 Ω*:cm*^2^ and VEGF ratio *<* 3) RPE cell functions in an implant. However, these measurement ranges can vary depending on the particular measurement approach (Chopstick or Endohm approach) and the types of polymer inserts [30,34].

To deliver RPE cell implants with high quality, one can quantify both shape-based and TER/VEGF-based criteria by analyzing segmented bright field images and by predicting TER/VEGF values. For performing segmentation and prediction analyses, Artificial Intelligence (AI) based models can be used. AI models can be divided into Traditional Machine Learning (TML) and Deep Learning (DL) based models. TML models depend on feature engineering while DL models perform feature engineering automatically but have higher modeling complexity. In addition, the use of these TML and DL models requires a preparation of annotated data, a model selection or its design, optimization of model parameters, engineering of relevant features, and so on. This motivates our work to explore the tradeoffs of TML and DL models to predict TER/VEGF/cell count of RPE cell implant. In this paper we used three prediction approaches using TML and DL models and these three prediction approaches are constructed directly or indirectly from calibrated bright field microscopy images with or without segmentation and feature extraction. The three prediction approaches are described as follows:

- **Approach 1 (indirect label prediction with segmentation and feature extraction):** Segment raw images into foreground (cells) and background using a Deep Learning model (DL_Seg), extract features from segmented cells, and predict the cell functions using machine learning (TML_Reg) model.
- **Approach 2 (direct label prediction):** Predict the cell functions directly from raw images using a Deep Learning (DL_Reg) model.
- **Approach 3 (indirect label prediction with feature extraction):** Extract features directly from raw images (per field of view) and predict the cell functions from the extracted features using Machine Learning (TML_Reg) model.

These three approaches have associated prediction accuracy, variability of accuracy with respect to implementation configurations, and overarching tradeoffs in terms of design complexity, human effort, and usability. The tradeoffs are summarized in Table 1. The modeling factors of the tradeoffs include (1) an overall complexity of modeling design, (2) number of modeling parameters, (3) global vs local optimization of modeling parameters, (4) level of effort required to create ground truth, (5) effort required to engineer the suitable features, (6) model transparency or interpretability, and (7) model generalizability. Our goal is to compare accuracies of the three approaches, quantify their accuracy variability across a few configurations, and explore the overarching tradeoffs between TML and DL based approaches when predicting TER, VEGF, and the number of cells per area, from the bright field microscopy images of RPE cell implants. In addition, we investigate the linked accuracy relationships between segmentation and the number of training samples, segmentation and cell features, and cell features and implant labels. The main contributions are:

- Comparison of tradeoffs between direct and indirect, TML and DL based approaches to RPE implant function predictions from microscopy images in order to minimize design complexity and human effort while maximizing the model accuracy and usability.
- Methodology for relating accuracies of pixel-, cell feature-, and implant label-level results in order to minimize the number of modeling steps.

Section 2 describes use of TML and DL models in biomedical imaging domain for cell segmentation, cell counting, drug discovery, nuclei detection, and cell function prediction tasks, but there are certain limitations in applying these models to a new dataset or a new task. The main limitations are limited data for training the models, complexities of designing a model, optimizing the model parameters, engineering the relevant features, and so on. Though DL models were successful in cell segmentation tasks, building such accurate models requires considerable amount of training data and creating such training data requires significant manual effort. On the other hand, unsupervised models do not require any training data but are less accurate and less robust to noise. Thus, there is a need to understand the tradeoffs between TML and DL models in the context of label prediction tasks (i.e., cell function prediction of RPE implants) with respect to the seven factors summarized in Table 1. This motivates our comparison of TML and DL based approaches for predicting the cell functions of RPE cell implants.

The paper is organized as follows: Section 2 presents related work. Section 3 describes the dataset and the TML and DL based approaches used for cell segmentation, feature extraction, and label prediction tasks, and the metrics used for the experimental analysis. Section 4 shows the experimental results and compares the approaches for cell function prediction task. Section 5 discusses the experimental results of the tradeoffs between TML and DL based approaches. Section 6 concludes the work.

## 2. Related work

Manually evaluating the quality of RPE cells is a tedious process because thousands of cells need to be detected and analyzed for their quality, shape, size, position etc. In the computer vision domain, there were traditional methods used for cell detection which incorporate thresholding, histogram equalization, median filtering, feature detection and other morphological operations that were applied in combination [20,23,24,41]. Rangel-Fonseca et al. proposed an unsupervised algorithm for RPE cell segmentation and quantifying the number of cells from segmented images [23]. Zafer et al. showed that a Support Vector Machine (SVM) model trained on multiple data types achieves very good accuracy in predicting the gene function but the SVM model is susceptible to noise [7]. Though machine learning models were widely used in the biomedical imaging, no single model is optimal for all types of problems [44].

Most of the machine learning based approaches used for cell segmentation were not generalizable and the performance of these approaches mainly depends on the relevant features extracted for a given task [32]. It was also shown that selecting the relevant features improved the classification of protein subcellular location images [10]. B. Ko et al. showed that a Random Forest (RF) classifier was more accurate in classifying white blood cells compared to other machine learning models. The RF model is good at classifying white blood cells with a small amount of training data using ensemble features [16]. Chuanxin Zou proposed a framework for sequence descriptor-based protein function prediction using a SVM model which exploits the protein properties to assist with feature selection [45]. In the past, many machine learning based algorithms have been used to build computational models for the prediction of protein structure classes such as SVM but prediction accuracy of TML methods was strongly affected by the sequence similarity of the training and testing datasets. Xiao-Juan Zhu et al. developed a SVM model to successfully predict the protein structural class with low similarity by choosing the selective features [43]. It was also shown that essential proteins were identified by integrating network topology and biological characteristics using Random walk based algorithm [21].

Finding relevant features is crucial for most TML based models. On the other hand, DL based models perform automatic feature engineering and have shown to be successful for many tasks in computer vision such as image classification, segmentation, and object detection [19,22,25]. Recently, there has been an increasing interest in applying DL based models to microscopy cell segmentation, detection, and cell counting tasks [3,26–28,37,38]. Hai-Cheng Yi has shown that DL models can learn high level features and the features extracted from a DL model were more accurate than other features for prediction of ncRNA-proteins [39]. It has also been shown that DL models were very accurate in predicting the locations of cells and their nuclei with 86% confidence [1]. Convolutional Neural Network (CNN) models were extensively applied to classification and segmentation of cells [18]. Zhiqiang Zhang et al. showed how deep learning technology can be used to predict and identify the functional units in DNA sequences, including replication domain, transcription factor binding site (TFBS), transcription initiation point, promoter, enhancer and gene deletion site [42].

Cell counting from microscopy images is an important task in many medical applications. This task was accomplished by segmenting images into contour masks using unsupervised and hybrid approaches [20,23]. Weidi Xie et al. proposed to estimate cell density without segmentation by a CNN based model applied to microsocpy images. In biomedical imaging, DL models outperform all traditional machine learning models in drug discovery applications as documented in a compariosn of TML and DL models by Alexander et al. [17]. Youyi Song compared DL with other TML models for cervical cancer cell segmentation and has shown that the DL model outperforms other TML models with 95% accuracy in detecting nucleus regions of cervical cancer cells [33]. For segmentation of cell nuclei in microscopy images, the DL model outperformed all the machine learning models [6].

## 3. Materials and methods

### 3.1. Materials

RPE implants were cultured and grown over a period of 155 days at the National Eye Institute (NEI), National Institute of Health (NIH). During this period of time, the implants were imaged by a bright field microscope. The cell implant functions were measured for TER and VEGF at multiple time points. The image acquisition was initiated after passing a stability imaging protocol and all images were converted to an absorbance pixel measurement(i.e. $-{\text{ log}}_{10}\left[\frac{\left(I-\text{Black}\right)}{\left(\text{White}-\text{Black}\right)}\right]$). Absorbance images were tiled into 256 256 images and pre-processed so that an image tile can be associated with implant-level TER and VEGF measurements. For each tile, ground truth segmentation of cells was obtained by manual segmentation. Each image tile was then associated with its ground truth cell count from the ground truth segmentation. Further details about the experimental design, sample preparation and imaging please refer to the article published recently in clinical investigation journal [46].

#### 3.1.1. Dataset used for RPE cell segmentation and prediction

As described earlier, all bright field microscopy images were converted to absorbance microscopy images. The number of absorbance images used for the segmentation task was 500 absorbance image tiles of size 256 × 256 acquired from RPE cell implants. These images were used to train the DL models for the segmentation task. Each image tile has a manually annotated ground-truth mask and corresponding TER, VEGF, and cell count value. The trained DL model is applied to segment 500 test absorbance images. For RPE cell function prediction, 500 test absorbance images are used.

#### 3.1.2. Performance metrics used for analysis

The three selected prediction approaches generate image segmentation, features extracted per cell or per field of view, and predicted regression values (TER, VEGF or cell count). These generated numerical results were evaluated using multiple metrics that are described below.

##### Pixel level metric:

We evaluated segmentation results of DL models at contour and region levels using the DICE similarity score [36]. DICE is defined as:

(1) $$\text{DICE}\left(G,P\right)=\frac{1}{n}\overset{N}{\underset{i=1}{\sum }}\frac{2\times G_{i}\cap P_{i}}{G_{i}\cup P_{i}}$$

where ‘*G*’ is a ground truth mask and ‘*P*’ is a predicted mask. The contour level DICE similarity score is calculated only by considering the foreground pixels (border pixels) and ‘*G*’ is considered as ground truth border pixel and ‘*P*’ is considered as predicted pixels corresponding to ground truth border pixel values. Coming to the region level DICE similarity score, it is calculated by considering the labels for each cell region where ‘*G*’ is considered as ground truth mask labels and ‘*P*’ is considered as predicted mask labels.

##### Feature level metric:

**Chi square** (*χ*^2^) distance is used to compute the feature histogram differences between the features extracted from absorbance images using ground truth masks and features extracted from absorbance images using predicted masks from the deep learning model. It is defined as:

(2) $$\chi^{2}\text{ distance}=\frac{1}{N}\overset{N}{\underset{i=1}{\sum }}\frac{{\left(G_{i}-P_{i}\right)}^{2}}{\left(G_{i}+P_{i}\right)}$$

##### Label level metric:

Root mean square error (RMSE) and *R*^2^ statistics are used to evaluate TER, VEGF, and cell count prediction accuracy

(3) $$\text{RMSE}=\sqrt{\frac{1}{N}\overset{N}{\underset{i=1}{\sum }}{\left(G_{i}-P_{i}\right)}^{2}}$$

where ‘*G*’ is considered as actual or ground truth *TER* and ‘*P*’ is considered as predicted *TER*.

(4) $$R^{2}=1-\frac{\sum_{i=1}^{N}{\left(G_{i}-P_{i}\right)}^{2}}{\sum_{i=1}^{N}{\left(G_{i}-\text{Mean}\left(P\right)\right)}^{2}}$$

where ‘*G*’ is considered as actual or ground truth cell function measurement (TER, VEGF, cell count) and ‘*P*’ is considered as predicted measurement of RPE implant.

### 3.2. Methods

**Fig. 1.**
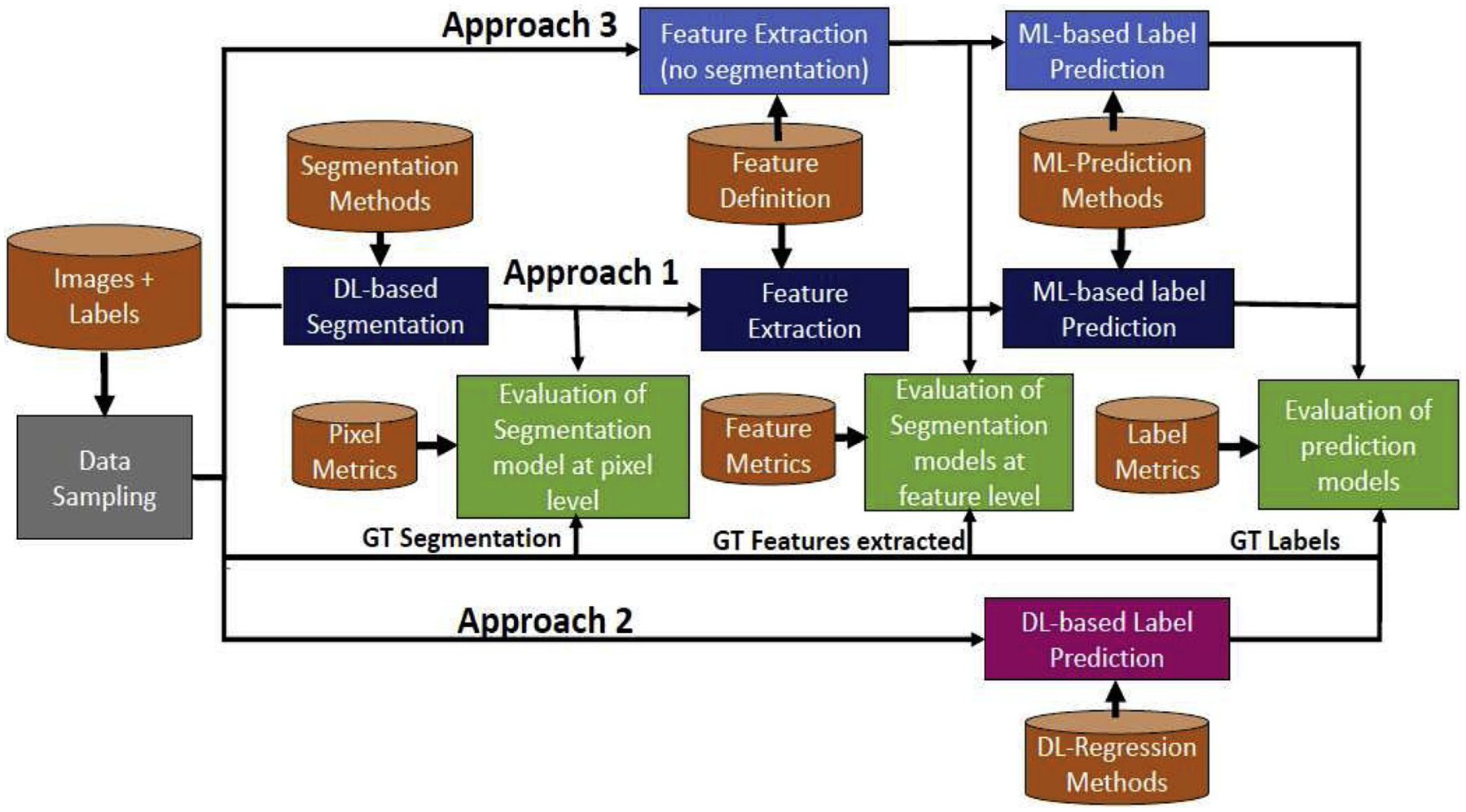
Data flow design of three approaches to cell function prediction. GT stands for ground truth, TML-Traditional Machine Learning.

Fig. 1 illustrates the three approaches used for solving the cell function prediction task. As shown in figure, each approach consists of specific models that are optimized against the ground truth using selected metrics. The optimization space that includes models, parameters, ground truth data, and optimization techniques is very large and therefore one must choose a feasible sub-space for model optimization. In this paper, we selected one DL model for segmentation (denoted as DL-Seg), one DL model for cell functional prediction (denoted as DL-Reg where Reg stands for regression), and five TML models for cell function prediction. In addition, we selected 37 features in the feature engineering step that include intensity, texture, and shape based descriptors. Finally, as discussed in Section 3.1.2. we chose three different metrics to evaluate the models at pixel-, feature-, and label-levels.The following sections describe all three approaches, the number of steps in each approach, implementations and configurations used for predicting the three RPE cell labels (TER, VEGF, and cell count).

#### 3.2.1. Approach 1: Indirect label prediction with segmentation and feature extraction

This approach consists of three steps: deep learning model for RPE cell segmentation task (DL_Seg), feature engineering and extraction of cell features from the segmented RPE absorbance images generated from DL_Seg model, and cell function prediction from cell features using a TML-based model. This pipeline is denoted as “DL_Seg+Extrac-t_Features + TML_Reg”. Table 2 shows the implementation steps and configurations for cell function prediction. Table 2 also includes libraries used for feature extraction and TML model analysis. As one can observe from the table, cell function prediction performance should depend on segmentation performance, types of extracted features, and a particular TML model used for prediction. The model design complexity of this approach is very high because we need to select a DL model for segmentation and a TML model for cell function prediction. The level of optimization required is very high because models need to be optimized at three different steps; segmentation, feature extraction, and cell function level comprising of global parameters involved in the DL model used for segmentation and local parameters that need to be optimized in the TML model. This approach is transparent by providing three accuracy probes, a DICE score for segmentation, *χ*^2^ difference for features, and RMSE for cell function prediction. Although this approach is transparent, it requires a lot of manual effort to create ground truth data for segmentation and to engineer the relevant features for TML prediction analysis.

##### Step 1: Segmentation

To segment RPE cell absorbance images into foreground (cells) and background pixels, we used a convolutional neural network (CNN) as a type of DL model with an encoder/decoder architecture. The encoder maps a given input image into a compact feature representation before the decoder maps the encoded feature representations to full input resolution feature maps for pixel-wise segmentation [4,26]. The model used in this paper is based on a U-Net CNN model architecture [26] and it is slightly modified in order to boost the model accuracy with transfer learning [40]. The encoder part of the U-Net architecture model is modified so that the coefficients of a model (called VGG16 or Oxford-Net) pretrained on the large ImageNet dataset [13] can be loaded into the encoder part of U-Net. After the U-Net model is initialized with the VGG16 coefficients, the entire U-Net model is refined and trained on RPE cell images. Table 1 in the supplementary section provides the details of the modified U-Net model architecture applied to the segmentation task.

The modified U-Net model is trained on RPE cell image tiles of size 256 × 256 and then accuracy is evaluated on 500 test images using two DICE similarity metrics (contour and region DICE).

##### Step 2: Feature Engineering

Once RPE absorbance images were segmented into contour masks, we applied a connected component analysis to obtain the cell regions. Given the cell regions the feature engineering step consists of selecting/constructing features, extracting features per region, and computing a histogram of features over all image tiles. The list of features used for the analysis are shown in Table 3. We extracted 37 features that are described as intensity, texture, and shape based features using the Web Image Processing Pipeline (WIPP) [5]. The WIPP system integrates multiple widely used feature extraction libraries and we used the ones implemented in Matlab. Finally, the histogram of all features was evaluated by using the *χ*^2^ feature histogram difference metric.

##### Step 3: Cell Function Prediction

As TER and VEGF measurements are continuous variables, we used regression models to predict the RPE cell function. The cell count is also considered as continuous measurements in order to reuse the same regression models for all three cell function labels. For all models, cell features are the independent variables and TER, VEGF and cell count are the dependent variables. We evaluated five TML models in our analyses as listed in Table 2. TML models are evaluated using the Weka machine learning library [14].

All TML models are trained on features extracted from 500 RPE cell images with a 66% training and 34% validation split to predict TER, VEGF and cell count image labels. Prediction accuracy is measured using the Root Mean Squared Error (RMSE) and *R*^2^ statistics as described in Section 3.1.2.

### 3.3. Approach 2: Direct label prediction

This approach consists of a single step, such as RPE cell function prediction from images. The implementation of this step uses the deep learning regression model denoted as “DL_Reg”. Table 4 lists the configuration details. The DL model architecture is similar to VGG16 with extra added fully connected layers and a number of filters used in convolutional layers.^1^ The DL model was trained and evaluated the same way as in Step 3 of the Approach 1 (i.e., 500 absorbance images, split 66% training and 34% validation, RMSE and *R*^2^ metrics).

### 3.4. Approach 3: Indirect label prediction with feature extraction

This approach consists of two steps, feature engineering and cell function prediction. First, features are extracted from RPE absorbance images and then the TML model is built to predict cell function from extracted features. This entire pipeline is denoted as “Extract_Features þ TML_Reg”. Table 5 shows the implementation steps. This approach does not depend on segmentation since features are extracted per field of view (FOV) because the prediction labels are collected at the FOV level. Since shape based features do not make sense in this case, only intensity and texture based features are extracted for cell function prediction.

## 4. Experimental results

The following sections discuss experimental evaluations of the three approaches and compare prediction accuracies.

### 4.1. Experimental setup

DL models used for RPE cell segmentation and cell function prediction are trained using NVIDIA Tesla P100 PCI-E 16 GB graphics processing units (GPUs) with CUDA 10.0 version. Deep learning models were implemented using Keras 2.0 tensorflow as backend. The DL segmentation model uses the Adam optimizer to minimize the binary cross-entropy loss. The model is trained for 8 gradient update steps corresponding to “300” epochs. Similarly, the DL regression model uses the Adadelta optimizer to minimize mean squared logarithmic error loss. The regression model is trained for 8 gradient update steps corresponding to “5000” epochs.

### 4.2. Accuracy comparison of three approaches

**Fig. 2.**
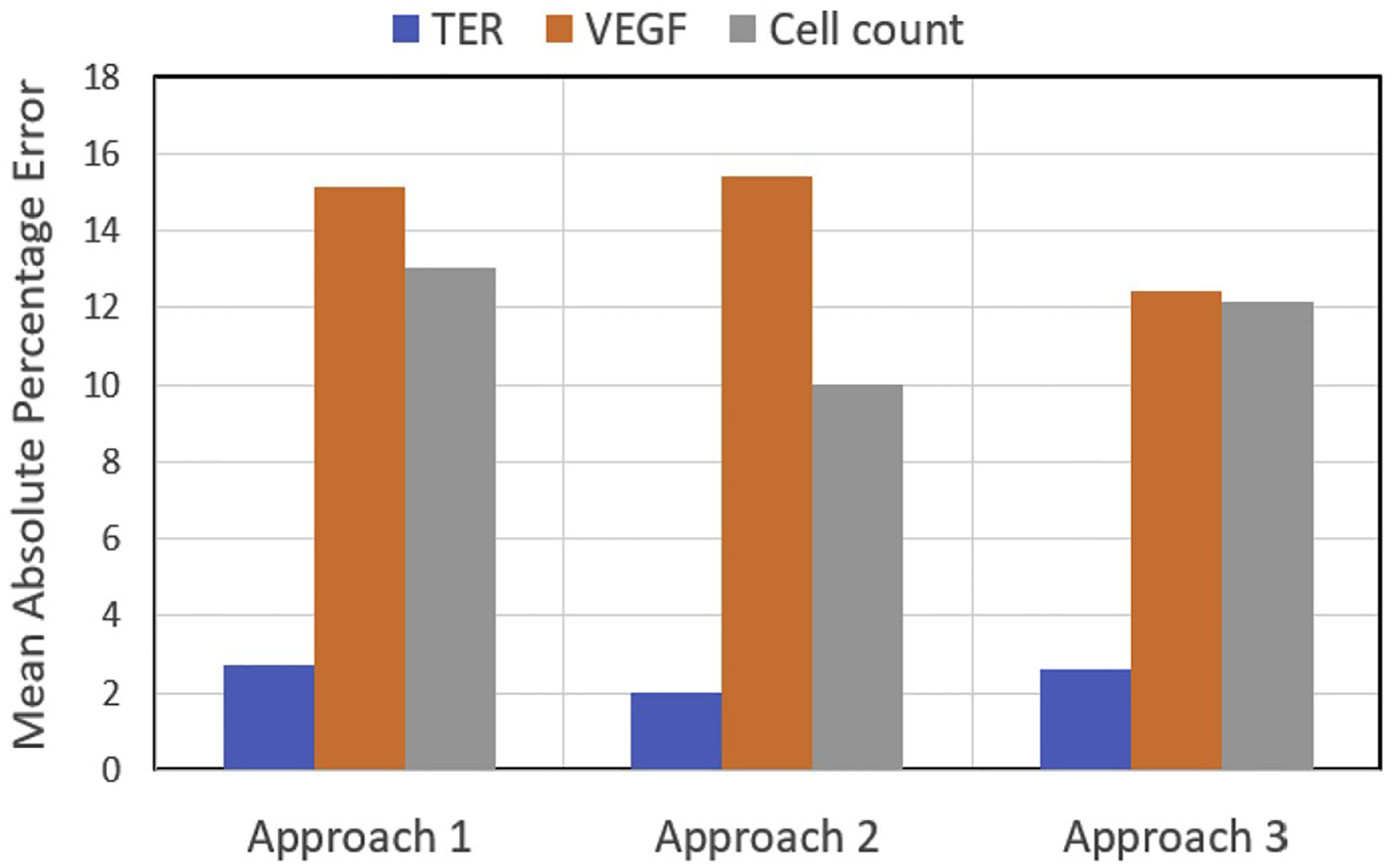
Mean Absolute Percentage Errors (MAPE) of three approaches for TER, VEGF, and Cell count predictions.

Table 6 shows the data ranges for TER, VEGF, and cell count measurements of RPE cell implants. Table 7 summarizes the accuracy comparison of cell function predictions using the three approaches. Figs. 1–3 in the supplementary section show predicted versus measured labels. Table 7 shows the mean errors of three approaches for cell function predictions and Fig. 2 gives the details about the percentage of errors relative to ground truth.

Based on Table 7 and the *R*^2^ values, Approach 2 is the only approach that achieves *R*^2^ values larger than 0.75 which could be considered as an indicator of a strong correlation between predicted values by the model and the ground truth values. Based on this criterion, model predictions using Approaches 1 and 3 do not show as strong correlations as Approach 2. We hypothesize that the weaker correlations are due to hand-crafted features in Approaches 1 and 3 since the features might not have been the most relevant for TER, VEGF, and cell count predictions.

Table 8 shows RMSE values from applying holdout and 5-fold cross validation to 500 images in the test dataset. As we can observe from Table 8 the results are very similar to each other and indicate robustness of the models to data sub-population.

**Fig. 3.**
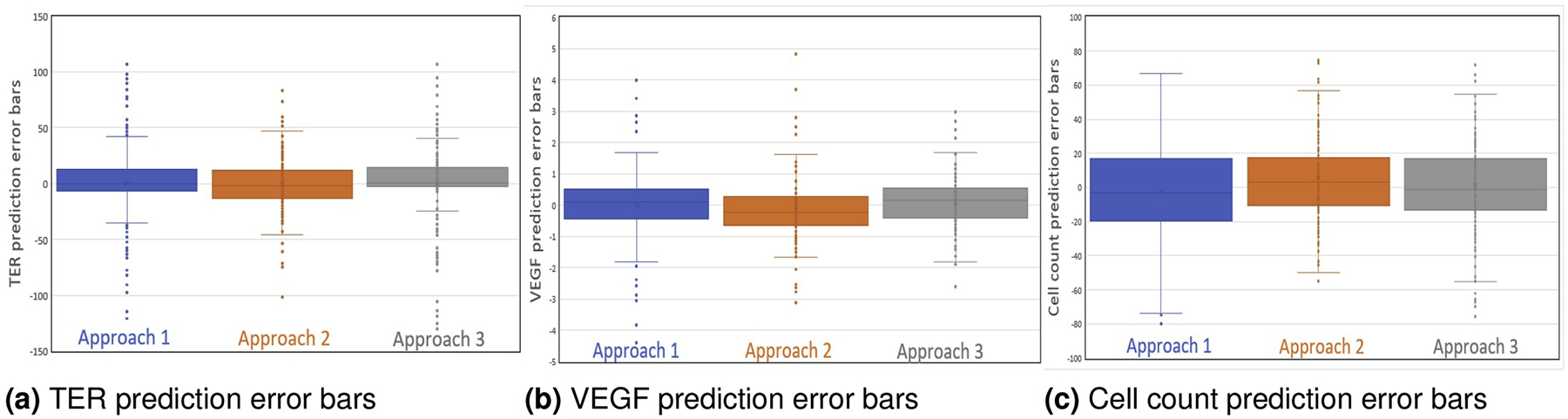
Box plots showing the distribution of errors while executing each approach to cell function predictions.

**Fig. 4.**
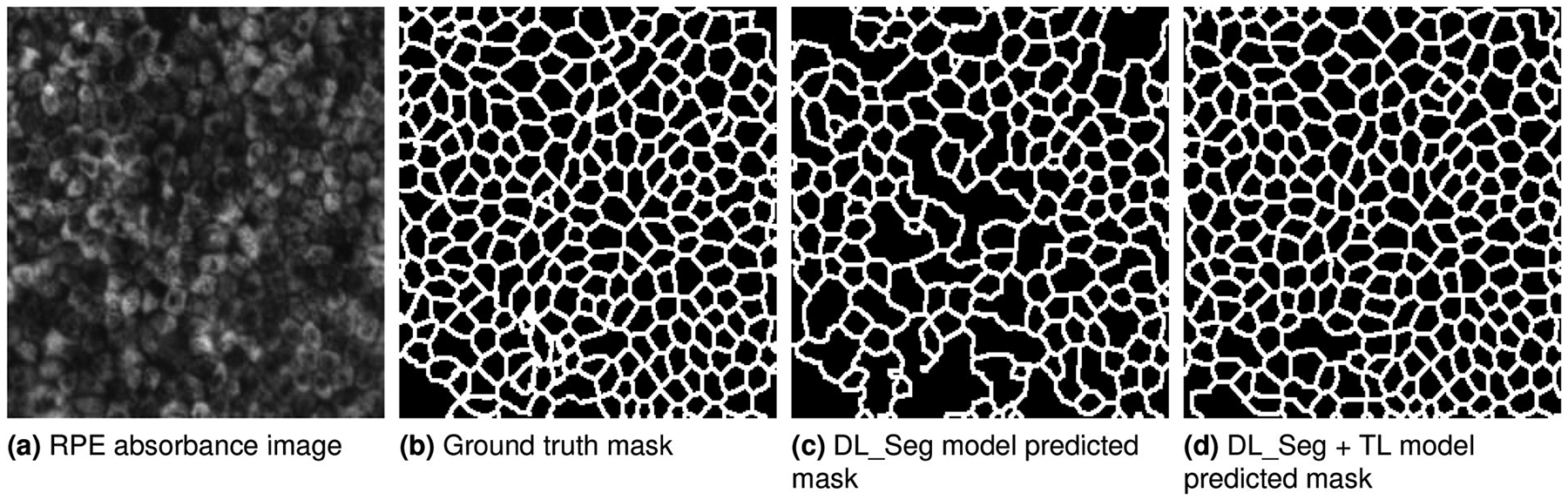
Visual comparison of segmentation results.

Fig. 3 illustrates the residuals plots for TER, VEGF, and cell count predictions of the three approaches. As we can see from Fig. 3, box plots overlap around the medians which are close to zero. The min and max ranges for Approaches 1 and 3 are slightly larger than the range for Approach 2. Approach 2 is symmetric around its median value for three predictions whereas Approaches 1 and 3 are skewed upwards or downwards indicating that these two approaches are overestimating or underestimating the cell function predictions. The spread of Approach 2 is much smaller (VEGF and Cell count) as compared to the other two approaches. Overall Approach 2, direct cell function prediction, is slightly more accurate as compared to the other two approaches. Figs. 4–6 in the supplementary section show residual error plots of the three label predictions. The error distribution is random indicating that the regression models are unbiased. Fig. 6 in the supplementary section shows the *t*-test comparison results for three approaches with 5% level of significance and 95% confidence. From the analysis, we can conclude that the three approaches are statistically similar in predicting cell function of RPE cell implants. Though these three approaches achieve similar accuracy, they have different trade-offs as summarized in Section 4.4.

### 4.3. Accuracy variability

We evaluated segmentation performance of the DL model with and without transfer learning, and cell function prediction using five TML models. Table 9 compares the results with and without transfer learning. The DL model with transfer learning improved the segmentation performance by 14% and 22% in terms of contour and region DICE scores respectively while reducing the cell count error by 12%. Thus, good segmentation leads to small error in cell count since cell count mainly depends on segmentation accuracy. Fig. 4a, b, 4c, and 4d illustrate a sample RPE absorbance image, ground truth segmentation and segmentation mask generated from DL models with and without transfer learning.

For the Approaches 1 and 3, Tables 10 and 11 compare the accuracy results of five different TML models for the cell function prediction task. The RF model outperformed the other TML models.

### 4.4. Tradeoffs of three approaches

Although accuracy comparisons of the three approaches yielded statistically similar performances, each of the approaches carries tradeoffs in terms of design complexity, human effort, and model usability as defined in Table 1. The model design complexity of Approach 2 is much simpler and it does not depend on segmentation and hand-crafted features. The main advantage of this method is that the model is optimized globally for cell function prediction as compared to Approach 1. This approach overcomes the manual effort required to create the ground truth and engineer the features for the prediction analysis. When analyzing Approach 3, though it does not depend on segmentation, its performance depends on optimization of feature engineering and on a choice of a TML model. This approach is less expensive in terms of model design, level of effort required to create the ground truth, the number of parameters involved, and the complexity involved in implementation. Table 13 summarizes the time comparison for inference on test images for the three approaches. Approach 2 is much faster than the other two approaches (of the order of milliseconds versus minutes).

We summarized the tradeoffs of all three approaches in Table 12 based on the seven factors defined in Table 1. If we order the three approaches based on the distance from the “ideal” attribute in Table 12, then the ranking from the smallest to the largest distance is: Approach 2, Approach 3, and Approach 1. Thus, from Tables 7 and 12, we concluded that Approach 2 has the potential to be the most accurate and effective approach in terms of the tradeoff factors.

Although based on ranking Approach 2 is the best approach for cell function prediction task, it has limitations in terms of model interpretability (transparency to a user) and computational requirements on exhaustive parameter optimization. Another limitation is the number of parameters involved in training the model. For example, the regression DL model has more parameters than the segmentation DL model because it contains fully connected layers and therefore it needs more images for training. One could reduce the number of parameters by optimizing a DL model over all architectures for a given regression task. However, this optimization is computationally expensive and is out of scope of this paper. In the future, we will plan to optimize DL models in each approach and select the most accurate DL model for segmentation and cell function prediction tasks.

### 4.5. Relationships between pixel-, feature-, and label-level accuracies

To understand the relationships between linked modeling accuracies of the steps in Approach 1, we designed a methodology as follows:

- Build multiple DL segmentation models for a varying number of training images.
- Apply DL segmentation models to segment 500 test images to obtain multiple sets of segmentation masks.
- Extract features from each set of segmentation masks.
- Predict cell functions from each set of features.
- Evaluate the accuracy of DL segmentation masks, feature histograms, and predicted labels using multiple metrics.

**Fig. 5.**
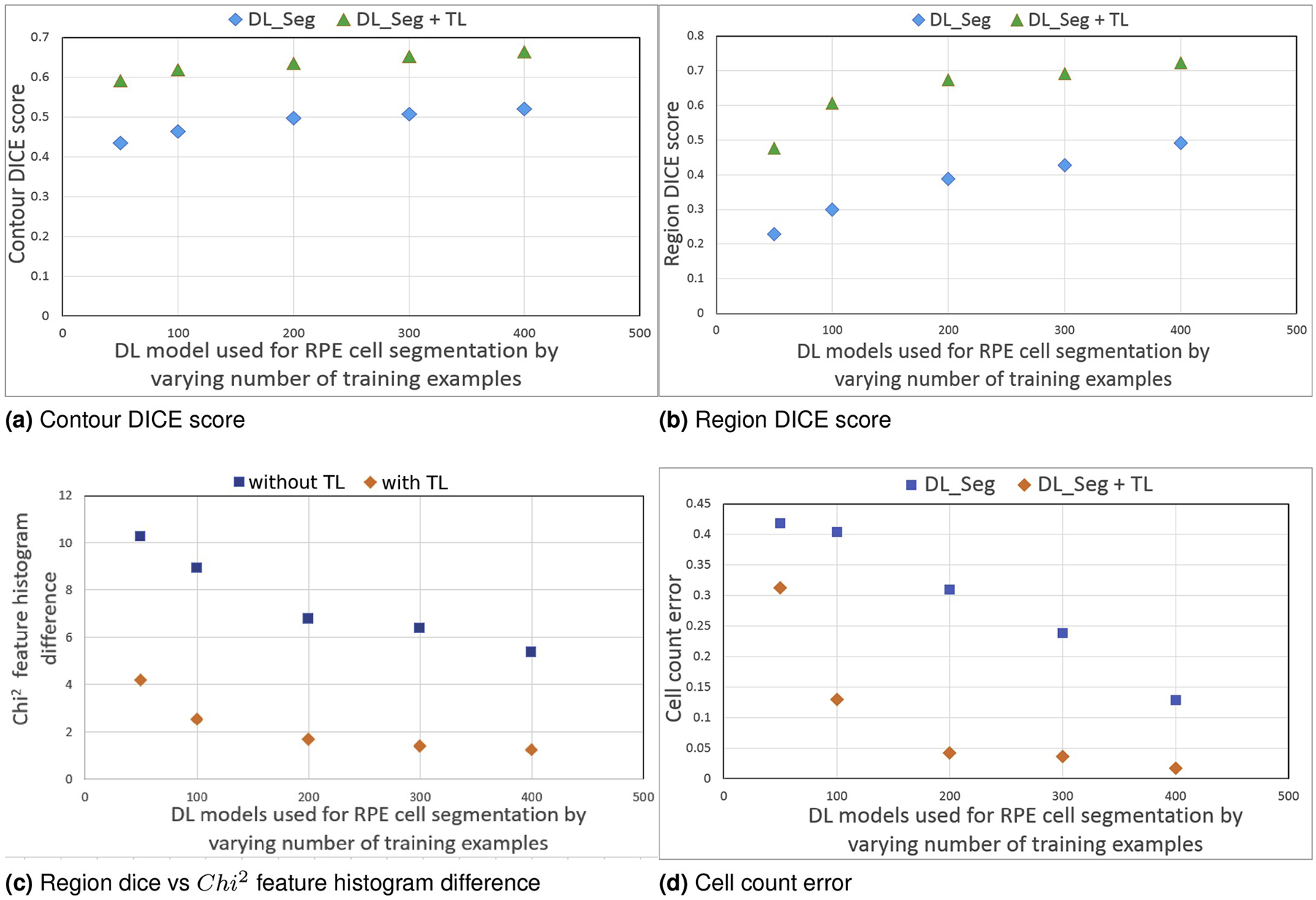
Segmentation accuracy comparisons of five DL models used for RPE cell segmentation task with and without transfer learning. DL_Seg model: Deep learning model used for RPE cell segmentation; TL: with transfer learning by adapting the VGG16 pretrained model weights.

In our study, we chose five DL segmentation models trained on 50, 100, 200, 300, and 400 training samples. These five models were tested on 500 test images. Fig. 5a, b, and 5d show the segmentation performances reported in terms of contour DICE, region DICE, and cell count error. As we increase the number of training examples, the segmentation accuracy increases and cell count error decreases. Fig. 5c shows how feature histogram difference changes with respect to segmentation accuracy. As expected, *χ*^2^ feature histogram difference and cell count error decrease as region DICE increases.

**Fig. 6.**
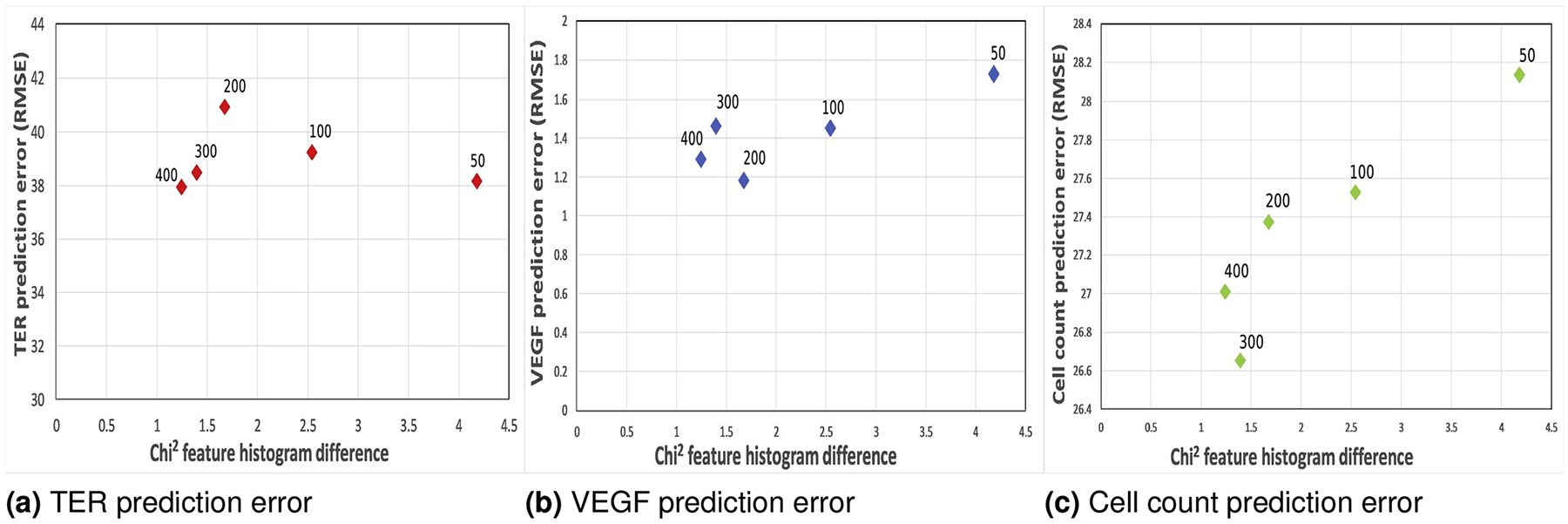
TER, VEGF, and cell count prediction errors (ranges of TER*<*127,1071*>*, VEGF*<*2.67,11.20*>*, cell count*<*33,298*>*) with respect to difference. The number next to each plotted data point refers to the number of training images.

Fig. 6 shows TER, VEGF, and cell count prediction errors with respect to *χ*^2^ feature histogram difference. If the segmentation step is important for cell function prediction, then the prediction error should decrease as *χ*^2^ feature histogram difference decreases. As it can be seen in Fig. 6, there is no correlation between feature histogram difference and TER and VEGF prediction accuracy but there is correlation with cell count. We hypothesize that TER and VEGF measurements are not sensitive to the microscopic image segmentation accuracy since they are tissue-level macroscopic measurements.

## 5. Discussions

From an experimental data view, our analysis is limited to a particular dataset which is made publicly available (600 GB). Additional experiments are needed to make correlations between good TER/VEGF levels and cell population distributions in a lerger variety of tissues.

From a parameter optimization view, this study covers a small portion of the search space formed by all possible implementations and configurations that can be constructed using the three common TML and DL based approaches. We showed that the three approaches can be statistically equivalent in terms of their prediction accuracy but are significantly different in terms of their design complexity, human effort, and model reusability. As the majority of the tradeoff factors was hard to quantify, the choice of an approach remains to be highly dependent on specific tasks and available resources. For example, the level of effort required for training data preparation might outweigh any other tradeoff factors. As summarized in Table 12, it is up to the user to select one of these approaches based on the application specific requirements.

All acquired data and the ground truth values are available to readers for browsing and downloading from here.^2^ The DL model for segmentation has been integrated into a software package WIPP which is available for downloading from here.^3^ The feature extraction tools are also available in WIPP.

## 6. Conclusions and future work

We presented cell function prediction results using three approaches leveraging TML and DL based modeling approaches. While the three prediction approaches have statistically similar accuracy performance, the direct TER/VEGF/cell count prediction method from images using a DL model was slightly more accurate than the other two indirect approaches using DL and TML models with intermediate segmentation and/or feature engineering steps.

Since each prediction approach had a large number of configuration parameters, we included in this study several illustrative results of configuration optimization. First, the image segmentation step was configured with and without transfer learning. The segmentation model with transfer learning improved segmentation accuracy by 25% as compared to the model without transfer learning while leveraging a pretrained model which was built on the ImageNet dataset. Next, the feature-based label prediction step was configured with five TML-based regression models. We reported the RF model to be the most accurate although less accurate than the direct DL-based approach.

We also compared TML and DL based approaches based on seven factors related to design complexity, human effort, and model reusability. Approach 2, direct label prediction, is ranked the highest with the drawbacks related to the lack of model transparency and a very large number of parameters to be optimized.

In addition, we illustrated a methodology for relating accuracies of intermediate pixel- and feature-level results to the ultimate label-level results. By using multiple-level evaluation metrics, we gained insights about (a) the sensitivity of each method to cell function prediction, (b) the relationships between accuracies achieved by each module within a method, and (c) the dependencies between prediction accuracy and segmentation accuracy. Based on such analyses, we showed that there is a relationship between the cell segmentation accuracy and the feature histogram dissimilarity (and the cell count error) but there is not a clear relationship between segmentation accuracy and cell function prediction accuracy.

Accuracy performance of Approaches 1 and 3 mainly depends on the feature engineering stage. Optimization over multiple feature selection methods may improve the cell function prediction performance. Future work may incorporate such additional optimizations as well as visualizations of DL models to provide useful insights about cell function prediction and cell segmentation tasks.

## Supplementary Material

1

## Figures and Tables

**Table 1:** Modeling factors considered to compare three approaches used for cell function prediction.

Type	Factors	Definition
Complexity	Complexity of modeling design	Exploration of plausible DL or TML model architectures for a given problem
	No. of modeling parameters	Number of parameters optimized during the training stage of the model
	Complexity of optimization	Number of independently optimized parameters with respect to DL & TML models
Effort	Training data preparation	Level of effort required to create ground truth
	Feature engineering	Effort required to engineer the suitable features
Usability	Model transparency or interpretability	Degree of interpretation of the resulting model coefficients
	Model generalizability	Degree of reusability in other domains

**Table 2:** Approach 1 implementation steps and configuration details. Abbreviations: WIPP- Web Image Processing Pipeline; RF-Random Forest regressor; SVR-Support Vector Regressor; LR-Linear Regressor; SLP-Single Layer Perceptron; MLP-Multi Layer Perceptron; RMSE-Root Mean Square Error.

Approach 1
**Step 1: Segmentation**
a) Implementation: Keras neural network library [11]
b) Configuration: Encoder & Decoder DL model [26]
i) Transfer learning
**Step 2: Feature Engineering**
a) Implementation: WIPP library [5]
b) Configuration: Intensity, Texture, Shape
i) Extracted per segment
ii) Selected manually
**Step 3: Cell Function Prediction**
a) Implementation: Weka library [14]
b) Configuration: Regression based models
i) RF, SVR, LR, SLP, & MLP

**Table 3:** List of features extracted for RPE cell function prediction.

Feature Name	Feature Type	Feature Name	Feature Type
Eccentricity	Spatial	Mean Intensity	Intensity
Extent	Spatial	Min Intensity	Intensity
Major Axis Length	Spatial	Max Intensity	Intensity
Minor Axis Length	Spatial	Standard Deviation	Intensity
Centroid	Spatial	Median Intensity	Intensity
Weighted Centroid	Spatial	Mode Intensity	Intensity
Area	Spatial	Skewness	Intensity
Perimeter	Spatial	Kurtosis	Intensity
Equivalent Diameter	Spatial	First Central Moment	Intensity
Orientation	Spatial	Contrast	Texture
Solidity	Spatial	Correlation	Texture
Bounding Box	Spatial	Energy	Texture
Euler Number	Spatial	Homogeneity	Texture
Filled Area	Spatial	Entropy	Texture
Convex Area	Spatial	Feret Diameter	Spatial
No. of Neighbors	Spatial	Border and Background	Spatial
		Neighbor	

**Table 4:** Approach 2 implementation steps and configuration details.

Approach 2
**Step 1: Cell Function Prediction**
a) Implementation: Keras neural network library [11]
b) Configuration: VGG16 CNN model [31]

**Table 5:** Approach 3 implementation steps and configuration details.

Approach 3
**Step 1: Feature Engineering**
a) Implementation: WIPP library [5]
b) Configuration: Intensity, Texture
i) Extracted per field of view (FOV)
ii) Selected manually
**Step 2: Cell Function Prediction**
a) Implementation: WEKA library [14]
b) Configuration: Regression based models
i) RF, SVR, LR, SLP, & MLP

**Table 6:** Range of values for TER, VEGF, and cell count measurements of RPE cell implants. FOV- per field of view. VEGF ratio- Measuring the VEGF secretion on basal side relative to apical side of the RPE cell monolayer (Basal side/Apical side).

Type of measurement	Min.value	Max.value
TER(Ω.*cm*^2^)	127	1071
VEGF ratio (Ba/Ap)	2.67	11.20
Cell count (per FOV)	33	298

**Table 7:** Comparison of three approaches used for cell function prediction. For Approaches 1 and 3, best machine learning model results are reported (Random forest regressor model performance is reported).

Approach	Error (mean)			Root Mean Squared Error (RMSE)			*R*^2^ statistics		
	TER	VEGF	Cell count	TER	VEGF	Cell count	TER	VEGF	Cell count
Approach 1	0.17	−0.006	−2.34	37.85	1.29	27.01	0.5253	0.794	0.6964
Approach 2	−0.59	−0.15	5.55	24.49	1.17	25.64	0.837	0.8442	0.7915
Approach 3	−0.265	0.097	1.00	38.48	0.90	27.31	0.5186	0.9095	0.6687

**Table 8:** Performance comparison of three approaches to cell function predictions evaluated using holdout and 5-fold cross validation methods. The TML based steps used Random Forest regressor model.

Approach	Root Mean Squared Error (RMSE)					
	Holdout validation			5-fold validation		
	TER	VEGF	Cell count	TER	VEGF	Cell count
Approach 1	37.85	1.29	27.01	40.63	1.20	25.97
Approach 2	24.49	1.17	25.64	27.87	1.14	23.11
Approach 3	38.48	0.90	27.31	38.20	0.97	26.37

**Table 9:** Segmentation accuracy comparison with and without transfer learning. DL_Seg model: Deep learning model used for RPE cell segmentation; TL: with transfer learning by adapting the VGG16 pretrained model weights.

Model	DICE score		Cell count error
	Contour	Region	
DL_Seg model	0.5209	0.4913	0.1290
DL_Seg model + TL	0.6638	0.7237	0.0171

**Table 10:** Performance comparison of TML regression models for cell function prediction using Approach 1.

Model	Root Mean Squared Error (RMSE)					
	Holdout validation			5-fold validation		
	TER	VEGF	Cell count	TER	VEGF	Cell count
LR	43.55	1.34	37.01	41.40	1.45	34.07
SVR	40.69	1.39	38.75	40.90	1.46	33.68
RF	37.85	1.29	27.01	40.63	1.20	25.97
SLP	58.94	2.00	39.44	53.41	1.85	40.96
MLP	48.85	1.32	33.00	48.71	1.20	30.74

**Table 11:** Performance comparison of TML regression models for cell function prediction using Approach 3.

Model	Root Mean Squared Error (RMSE)					
	Holdout validation			5-fold validation		
	TER	VEGF	Cell count	TER	VEGF	Cell count
LR	46.66	1.18	40.81	48.02	1.29	38.65
SVR	43.98	1.27	36.52	48.92	1.29	35.26
RF	38.48	0.90	27.31	38.20	0.97	26.37
SLP	44.95	1.60	37.51	53.64	1.49	36.60
MLP	34.55	0.5707	34.55	38.50	0.72	33.54

**Table 12:** Qualitative tradeoffs of the three approaches applied to RPE cell prediction problem. The labels “low”, “medium” and “high” are qualitative values and are assigned based on comparative assessments with respect to ideal values.

Factors	Approach 1	Approach 2	Approach 3	Ideal
Complexity of modeling design	high (2)	low (0)	medium	low (0)
No. of modeling parameters	medium (1)	high (2)	low (0)	low (0)
Complexity of optimization	high (2)	low (0)	medium (1)	low (0)
Training data preparation	high (2)	low (0)	low (0)	low (0)
Feature engineering	manual (2)	automatic (0)	manual (2)	automatic (0)
Model transparency	high (2)	low (0)	medium (1)	high (2)
Model generalizability	medium (1)	high (2)	low (0)	high (2)

**Table 13:** Qualitative comparison of inference times for three approaches.Times are measured in milliseconds (ms), minutes (min).

Approach	Test time	Approximate time	Speed
1	DL_Seg + FE + TML	ms + min + min	low
2	DL_Reg	ms	high
3	FE + TML	min + min	low

## Footnotes

1 See Table 2 in the supplementary section.

2 https://isg.nist.gov/deepzoomweb/data/RPEimplants.

3 https://isg.nist.gov/deepzoomweb/software/wipp.
