# Supplementary material for: Comparison of Artificial Intelligence based approaches to cell function prediction
Source: Inform Med Unlocked. Author manuscript; Available in PMC 2020 Aug 27. (PMC7450761)
Supplement: 1 [file NIHMS1579969-supplement-1.pdf]

Supplementary Information

Layer (type)	Output Shape	Param
InputLayer	(256,256,1)	0
block1-conv1	(256,256,64)	640
batch-norm-1	(256,256,64)	256
conv1-pool	(128,128,64)	0
block2-conv1	(128,128,128)	512
batch-norm-2	(128,128,128)	512
conv2-pool	(64,64,128)	0
block3-conv1	(64,64,256)	295168
batch-norm-3	(64,64,256)	1024
conv3-pool	(32,32,256)	0
block4-conv1	(32,32,512)	1180160
batch-norm-4	(32,32,512)	2048
dropout-1	(32,32,512)	0
conv4-pool	(16,16,512)	0
up-sampling2d-1	(32,32,512)	0
conv2d-1	(32,32,512)	1049088
conv2d-2	(32,32,512)	2359808
batch-norm-5	(32,32,512)	2048
up-sampling2d-2	(64,64,512)	0
conv2d-3	(64,64,256)	524544
conv2d-4	(64,64,256)	590080
batch-norm-6	(64,64,256)	1024
up-sampling2d-3	(128,128,256)	0
conv2d-5	(128,128,128)	131200
conv2d-6	(128,128,128)	147584
batch-norm-7	(128,128,128)	512
up-sampling2d-4	(256,256,128)	0
conv2d-7	(256,256,64)	32832
conv2d-8	(256,256,64)	0
conv2d-9	(2456,256,2)	1154
conv2d-10	(256,256,1)	2

Table 1: Deep learning model architecture used for segmentation of RPE cell absorbance images into contour masks. The encoder part of the model is similar to VGG16 model so that VGG16 pretrained weights can be directly loaded to the encoder part of the model for segmentation task.

Layer (type)	Output Shape	Param
InputLayer	(256,256,1)	0
block1-conv1	(256,256,32)	320
batch-norm-1	(256,256,32)	128
conv1-pool	(128,128,32)	0
block2-conv1	(128,128,64)	18496
batch-norm-2	(128,128,64)	256
conv2-pool	(64,64,64)	0
block3-conv1	(64,64,128)	73856
batch-norm-3	(64,64,128)	512
conv3-pool	(32,32,128)	0
block4-conv1	(32,32,256)	295168
batch-norm-4	(32,32,256)	1024
conv4-pool	(16,16,256)	0
block5-conv1	(16,16,512)	1180160
batch-norm-5	(16,16,512)	2048
conv5-pool	(8,8,512)	0
Dense-1	32	1048608
dropout-1	32	0
Dense-2	16	528
dropout-2	16	0
Dense-3	1	17

Table 2: Deep learning model architecture used for TER, VEGF, cell count predictions directly from the RPE absorbance images. This model is referred to as DL_Reg model which is different from DL_Seg model.

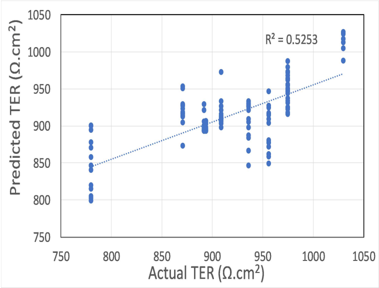

(a) Approach 1

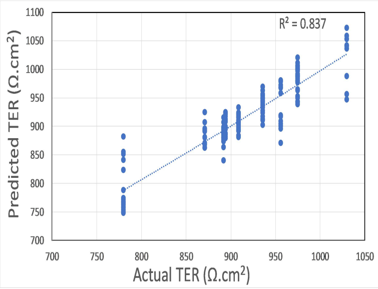

(b) Approach 2

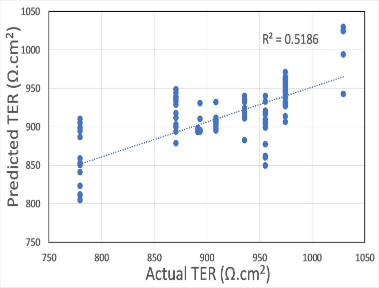

(c) Approach 3

Figure 1: Predicted values vs actual measurements of TER cell function predictions using three approaches

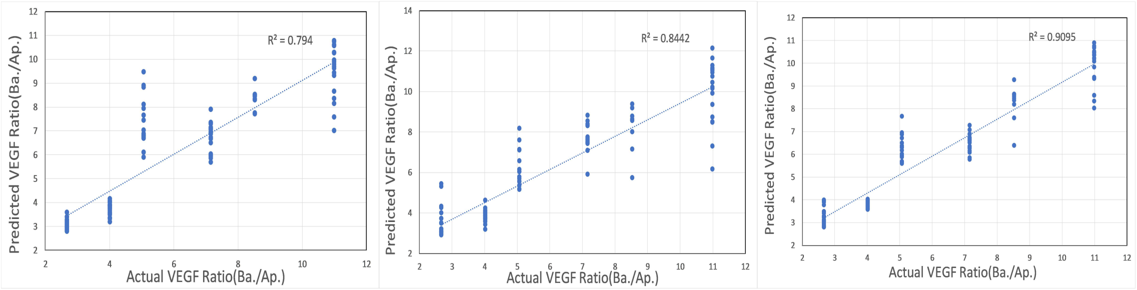

(**a**) Approach 1 (**b**) Approach 2 (**c**) Approach 3

Figure 2: Predicted values vs actual measurements of VEGF cell function predictions using three approaches

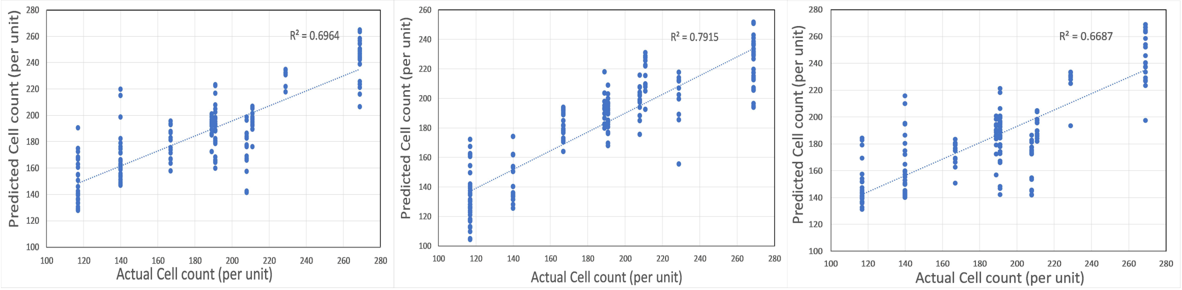

(**a**) Approach 1 (**b**) Approach 2 (**c**) Approach 3

Figure 3: Predicted values vs actual measurements of cell count predictions using three approaches

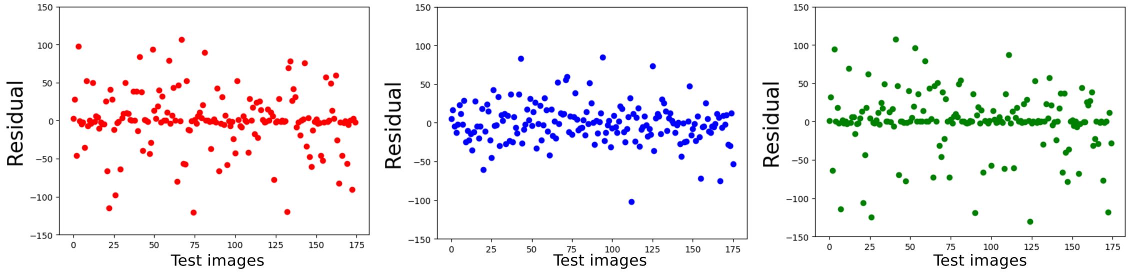

(**a**) Approach 1 (**b**) Approach 2 (**c**) Approach 3

Figure 4: TER prediction residual error plots for three approaches

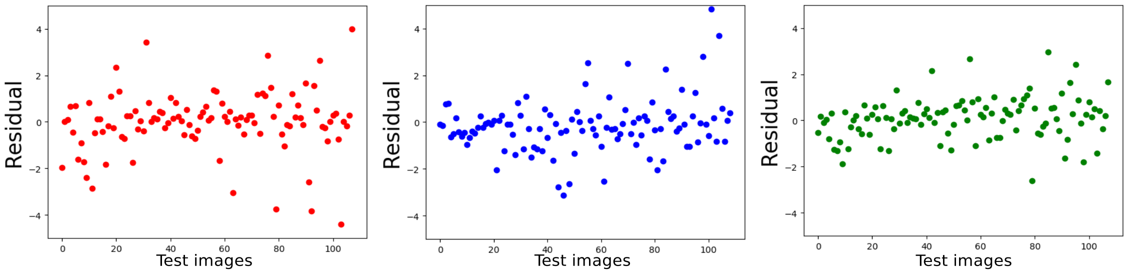

(a) Approach 1 **(b)** Approach 2 **(c)** Approach 3

Figure 5: VEGF prediction residual error plots for three approaches

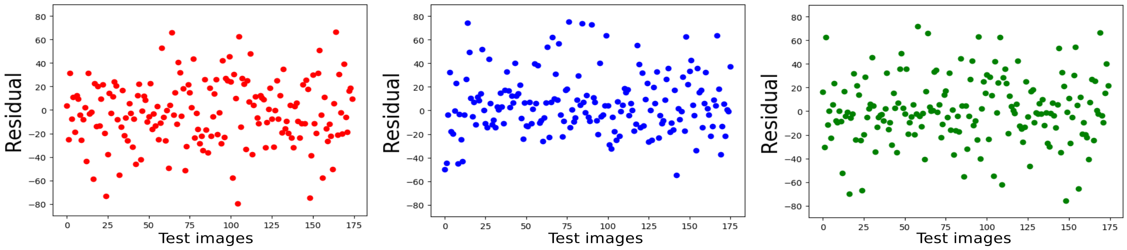

(a) Approach 1 **(b)** Approach 2 **(c)** Approach 3

Figure 6: Cell count prediction residual error plots for three approaches

TER Predictions: t-Test: Two-Sample Assuming Unequal Variances								
**Approach 2 vs 1**			**Approach 2 vs 3**			**Approach 1 vs 3**		
	Variable	*Variable 2*		*Variable*	*Variable 2*		*Variable*	*Variable 2*
Mean	913.2	910.4784	Mean	913.2	911.0072	Mean	910	911.0072
Variance	3978	1451.149	Variance	3978	1218.949	Variance	1451	1218.949
Observations	175	175	Observations	175	175	Observations	175	175
Hypothesized Mean D	0		Hypothesized Mea	0		Hypothesized Mea	0	
df	286		df	271		df	345	
t Stat	0.483		t Stat	0.397		t Stat	-0.14	
P(T<=t) one-tail	0.315		P(T<=t) one-tail	0.346		P(T<=t) one-tail	0.45	
t Critical one-tail	1.65		t Critical one-tail	1.65		t Critical one-tail	1.65	
P(T<=t) two-tail	**0.629**		P(T<=t) two-tail	**0.692**		P(T<=t) two-tail	**0.89**	
t Critical two-tail	1.968		t Critical two-tail	1.969		t Critical two-tail	1.97	

(a) TER prediction statistics

VEGF Prediction: t-Test: Two-Sample Assuming Unequal Variances								
**Approach 2 vs 1**			**Approach 2 vs 3**			**Approach 1 vs 3**		
	Variable 1	*Variable 2*		*Variable 1*	*Variable 2*		*Variable 1*	*Variable 2*
Mean	6.111629	6.058241	Mean	6.11163	5.954954	Mean	6.05824	5.954954
Variance	6.928325	6.146358	Variance	6.92832	5.944242	Variance	6.14636	5.944242
Observations	108	108	Observation	108	108	Observation	108	108
Hypothesized Mean Di	0		Hypothesize	0		Hypothesize	0	
df	213		df	213		df	213	
t Stat	0.15344		t Stat	0.45381		t Stat	0.3087	
P(T<=t) one-tail	0.439098		P(T<=t) one-	0.32521		P(T<=t) one-	0.37893	
t Critical one-tail	1.652039		t Critical one	1.65204		t Critical one	1.65201	
P(T<=t) two-tail	**0.878197**		P(T<=t) two-	**0.65042**		P(T<=t) two-	**0.75785**	
t Critical two-tail	1.971164		t Critical two	1.97116		t Critical two	1.97111	

(b) VEGF prediction statistics

Cell count prediction: t-Test: Two-Sample Assuming Unequal Variances								
**Approach 2 vs 1**			**Approach 2 vs 3**			**Approach 1 vs 3**		
	Variable 1	*Variable 2*		*Variable 1*	*Variable 2*		*Variable 1*	*Variable 2*
Mean	181.87	186.891	Mean	181.87	183.54	Mean	186.89	183.54
Variance	1348.74	1035.014	Variance	1348.74	1241.21	Variance	1035.01	1241.21
Observations	175	175	Observatic	175	175	Observatio	175	175
Hypothesized Mean Difference	0		Hypothesi	0		Hypothesiz	0	
df	342		df	347		df	345	
t Stat	-1.36		t Stat	-0.43		t Stat	0.93	
P(T<=t) one-tail	0.09		P(T<=t) on	0.33		P(T<=t) one	0.18	
t Critical one-tail	1.65		t Critical o	1.65		t Critical on	1.65	
P(T<=t) two-tail	**0.17**		P(T<=t) tw	**0.66**		P(T<=t) two	**0.35**	
t Critical two-tail	1.97		t Critical t	1.97		t Critical tw	1.97	

(c) Cell count prediction statistics

Figure 7: T-test statistics to show the statistical similarity between three approaches in predicting the cell function
